# Opposing Ageing-Related Transcriptomic Profiles Distinguish Opioid Exposure Liability From Dependence Liability

**DOI:** 10.7759/cureus.111258

**Published:** 2026-06-21

**Authors:** Ngo Cheung

**Affiliations:** 1 Psychiatry, Cheung Ngo Medical Limited, Hong Kong, HKG

**Keywords:** addiction psychiatry, age and ageing, clinical genomics, opioid use disorders, twas

## Abstract

Opioid use disorder (OUD) involves separable stages of exposure and dependence, but many genetic studies have treated these liabilities as a single phenotype. In this study, transcriptome-wide association study (TWAS) methods were applied to the Psychiatric Genomics Consortium Substance Use Disorders 2020 opioid meta-analysis summary statistics to examine ageing-related gene pathways across brain tissues. Genetic liability for opioid exposure was strongly associated with reduced predicted expression of complement components and synaptic-pruning genes, together with negative signals in astrocyte and senescence-related gene sets. By contrast, liability for dependence among opioid-exposed individuals showed positive enrichment in AMP-activated protein kinase-mechanistic target of rapamycin nutrient-sensing pathways, mitochondrial oxidative phosphorylation, especially DLD, and selected nicotinamide adenine dinucleotide/sirtuin pathways, accompanied by presynaptic and DNA repair-associated signals. Many ageing-related gene sets showed opposing directional profiles between exposure and dependence contrasts, indicating that these stages are associated with partially antagonistic transcriptomic profiles. Gene-level analyses identified influential drivers and directional sign-flips, including CASP7, TERF2, STX1A, and ADCY6, which further supported the stage-specific pattern. A dedicated post-hoc robustness inventory was performed, including threshold variation, family-wise false discovery rate pooling, tissue-specific decomposition, gene-set overlap analysis, major histocompatibility complex/complement flagging, and leave-one-out influence analysis. In this inventory, 131 of 4913 pooled statistical tests survived global Benjamini-Hochberg false discovery rate correction, and 100 of 102 evaluable pathway-profile entries satisfied an opposing exposure-versus-dependence criterion. These findings suggest that opioid exposure and opioid dependence are biologically distinguishable at the level of ageing-related brain gene expression. The results nominate stage-specific risk modules and biomarker hypotheses that may inform future precision approaches to OUD prevention and treatment while remaining hypothesis-generating until validated in functional and longitudinal studies.

## Introduction

Opioid use disorder (OUD) remains a major public health problem and contributes substantially to morbidity, mortality, and healthcare burden worldwide. Genetic factors account for a meaningful proportion of risk, with heritability estimates for opioid dependence generally ranging from 40% to 60% [1,2]. Large-scale genome-wide association studies (GWAS) have begun to identify common genetic variants associated with OUD, yet translating these associations into biological mechanisms remains difficult. The Psychiatric Genomics Consortium Substance Use Disorders (PGC-SUD) workgroup released a 2020 opioid meta-analysis that separated three key phenotypic contrasts: opioid dependence cases versus opioid-exposed controls, opioid dependence cases versus opioid-unexposed controls, and opioid-exposed controls versus opioid-unexposed controls [3]. This decomposition is valuable because standard case-control studies can blur genetic liability for exposure, such as opportunity, medical access, or risk-taking, with genetic liability for progression to dependence once exposure has occurred.

Conventional GWAS have important limitations for this question. Many analyses collapse exposure and dependence into a single case definition, which makes it difficult to determine whether associated variants influence the likelihood of trying opioids, the reinforcing effects of initial use, or the neuroadaptations that support compulsive use and withdrawal. GWAS also identifies genomic loci rather than the specific genes or pathways through which variants act. Because many risk variants lie outside protein-coding regions, functional interpretation often requires additional data layers, including expression quantitative trait loci that connect genetic variation to gene expression in relevant tissues.

Transcriptome-wide association studies (TWAS) address this problem by integrating GWAS summary statistics with reference transcriptome data. These methods estimate genetically predicted gene expression and test whether predicted expression is associated with a trait of interest [4,5]. When applied to brain tissues from the Genotype-Tissue Expression (GTEx) project, TWAS can identify genes whose predicted expression in reward, limbic, and cortical regions is associated with opioid phenotypes [6]. In the present study, ageing-related pathways were selected as the focus because several core ageing processes overlap with known addiction biology. These include chronic low-grade inflammation, mitochondrial dysfunction, nicotinamide adenine dinucleotide (NAD) metabolism and sirtuin activity, altered synaptic plasticity, and cellular senescence [7-10]. Chronic opioid exposure has also been associated with neuroinflammatory changes, oxidative stress, mitochondrial impairment, and disrupted synaptic homeostasis [11-13]. These overlaps raise the possibility that genetic variation affecting ageing-related processes may shape liability at different stages of opioid involvement.

Few studies have separated genetic liability for opioid exposure from liability for dependence conditional on exposure at the level of brain gene expression or pathway enrichment. Existing transcriptomic work has often focused on chronic opioid exposure in model systems or postmortem tissue from individuals with long-standing OUD, leaving the genetic architecture of the transition from exposure to dependence less well characterized. The present study therefore applied TWAS followed by targeted enrichment analysis of ageing-related gene sets to the PGC-SUD 2020 opioid summary statistics. The aims were to identify ageing-related pathways enriched in exposure and dependence contrasts, examine individual genes and their directionality across phenotypes, and generate translational hypotheses for risk stratification, biomarker development, and stage-specific intervention.

Objectives and hypotheses

The primary objective was to compare the directionality and enrichment profiles of ageing-related TWAS signals between opioid exposure liability, defined as opioid-exposed controls versus opioid-unexposed controls (EXP-UNX), and dependence liability conditional on exposure, defined as opioid dependence cases versus opioid-exposed controls (DEP-EXP). Secondary objectives were to examine tissue specificity and cross-tissue aggregation, identify influential genes and directional sign-flips, quantify profile concordance and discordance using formal statistical tests, and evaluate robustness to threshold choice, multiple testing, gene-set overlap, and major histocompatibility complex (MHC)/complement-driven signals. The central hypothesis was that opioid exposure liability and dependence liability would show partially opposing ageing-related transcriptomic profiles rather than a single monotonic continuum.

The analyses showed that genetic liability for opioid exposure was associated with reduced predicted expression of complement components and synaptic-pruning genes. In contrast, liability for dependence among exposed individuals was associated with positive enrichment of AMP-activated protein kinase (AMPK)-mechanistic target of rapamycin (mTOR) nutrient-sensing pathways, mitochondrial oxidative phosphorylation, and selected NAD/sirtuin pathways. Many ageing-related gene sets showed opposing profiles between exposure and dependence contrasts, suggesting that these two stages of opioid involvement differ not only in degree but also in directionally distinct transcriptomic organization.

## Materials and methods

GWAS summary statistics

Publicly available summary statistics from the PGC-SUD 2020 release were used for all analyses [3]. The meta-analysis included three primary opioid phenotypes across European-ancestry, African-ancestry, and trans-ancestry cohorts. These were opioid dependence cases versus opioid-exposed controls, referred to here as oud oex or DEP versus EXP; opioid dependence cases versus opioid-unexposed controls, referred to as oud uex or DEP versus UNX; and opioid-exposed versus opioid-unexposed controls, referred to as oex uex or EXP versus UNX. Phenotype definitions followed the Diagnostic and Statistical Manual of Mental Disorders, Fourth Edition, criteria for opioid dependence as implemented in the PGC-SUD 2020 opioid meta-analysis, with opioid exposure defined as lifetime opioid exposure including licit or illicit opioid use [3]. Sample sizes differed by ancestry and phenotype but collectively exceeded 41,000 individuals. The main analyses focused primarily on trans-ancestry and European-ancestry results, with ancestry-specific checks used as sensitivity analyses. Quality control, imputation, and meta-analytic procedures followed the original PGC-SUD publication [3].

TWAS pipeline

TWAS was performed using S-PrediXcan, an extension of the PrediXcan framework that uses GWAS summary statistics rather than individual-level genotype and phenotype data [4,14]. S-PrediXcan estimates associations between genetically predicted gene expression and a phenotype by using precomputed prediction models trained in reference transcriptome datasets. GTEx v8 brain tissue models were used, including the anterior cingulate cortex BA24, caudate basal ganglia, nucleus accumbens basal ganglia, putamen basal ganglia, amygdala, hippocampus, frontal cortex BA9, and cerebellum [6]. These regions were chosen before analysis because of their relevance to reward processing, emotional regulation, cognitive control, and addiction-related neurobiology. Prediction models were restricted to genes with cross-validated prediction performance of R² greater than 0.01 and training data p-value less than 0.05, consistent with commonly used GTEx/MetaXcan model-performance filtering. For each gene-tissue pair, a TWAS Z-score was calculated to represent the association between genetically predicted expression and each opioid phenotype. Analyses used the publicly available S-PrediXcan software and GTEx prediction weights from the MetaXcan/PredictDB framework.

For cross-tissue analyses, a single "Global" gene-level Z-score was computed for each gene and opioid contrast by combining available tissue-specific S-PrediXcan Z-scores using an unweighted Stouffer procedure: tissue Z-scores were summed and divided by the square root of the number of available tissue models for that gene. This approach was used to obtain one aggregate gene statistic per contrast before gene-set enrichment and profile analyses. Correlations among GTEx brain tissues were not explicitly modelled, and this is treated as a limitation. Tissue-specific analyses were therefore retained as a sensitivity and interpretive check rather than as independent confirmation of the Global signal.

Gene-set enrichment analysis

To identify coordinated biological signals, competitive gene-set enrichment was conducted using a multi-gene-set mode implemented in a custom Python analysis pipeline. A total of 54 ageing-related gene sets were tested. A focused collection of ageing-related gene sets was curated from the Molecular Signatures Database (MSigDB), Reactome, Gene Ontology, HALLMARK, Kyoto Encyclopedia of Genes and Genomes (KEGG), WikiPathways, and the SenMayo senescence-associated secretory phenotype collection [15-21]. These sets were selected to capture core ageing-related processes, including cellular senescence, mitochondrial function and oxidative phosphorylation, NAD metabolism and sirtuin activity, telomere maintenance, apoptosis, complement and innate immune signaling, synaptic plasticity, glutamatergic transmission, and circadian regulation. Gene-set membership was derived from MSigDB and curated pathway databases, followed by manual review to remove sets that were overly broad or obviously irrelevant. Pairwise Jaccard overlap was also quantified in the post-hoc sensitivity package; the largest overlap was observed between REACTOME CELLULAR SENESCENCE ALL and REACTOME SENESCENCE ALL, which were effectively duplicate Reactome-derived sets.

Gene-set names are reported using their source database terminology when appropriate. In MSigDB naming, terms such as HALLMARK, REACTOME, KEGG, GO, and WP indicate the source collection from which the gene set was obtained. The suffix ALL denotes the complete curated gene set from that collection rather than a subset selected after analysis. For readability, these database labels are accompanied in the text and table legends by plain-language descriptions. For example, MOOTHA MITOCHONDRIA ALL refers to a curated mitochondrial gene set originally derived from mitochondrial biology studies, HALLMARK OXIDATIVE PHOSPHORYLATION ALL refers to the Hallmark oxidative phosphorylation gene set, and REACTOME ENERGY DEPENDENT REGULATION OF MTOR BY LKB1 AMPK ALL refers to Reactome genes involved in energy-dependent regulation of mTOR complex 1 signaling by the liver kinase B1-AMPK pathway.

For each phenotype-tissue combination, Stouffer's Z-score was calculated as a combined measure of enrichment direction and magnitude across genes within each set. Wilcoxon signed-rank statistics were computed to test whether gene-set TWAS Z-scores differed systematically from zero. Permutation-based p-values were generated using 10,000 permutations by randomly sampling gene sets of equivalent size from the genome-wide TWAS Z-score distribution without replacement. This empirical competitive null controlled for gene-set size but did not explicitly preserve linkage disequilibrium or gene-gene correlation; correlated TWAS signals and shared expression regulation are therefore noted as limitations. A nominal threshold of p-value less than 0.05 was used for both permutation and Wilcoxon tests, and false discovery rate correction was applied across tested sets. To limit unstable estimates, only sets with at least five genes with non-missing TWAS statistics were retained for primary interpretation.

Tissue-level enrichment was used as an exploratory regional decomposition of the Global signal. Because the tissue-level sensitivity run did not compute permutation p-values, tissue-specific rows are reported with Wilcoxon p-values only and are labelled exploratory.

Differential, proximity, and concordance analyses

Pairwise comparisons were performed between the three opioid phenotypes: oex uex versus oud oex, oex uex versus oud uex, and oud oex versus oud uex. Mann-Whitney U tests were applied to the distribution of TWAS Z-scores within each gene set, and paired Wilcoxon signed-rank tests were used when overlapping genes allowed paired comparisons. Effect sizes were measured using rank-biserial correlation and Cohen's d. For tabular reporting, directional effect sizes were aligned so that positive values indicate higher or more positive Z-score ranks in the first contrast listed and negative values indicate lower or more negative ranks in the first contrast listed. Profile similarity across phenotypes was evaluated using Pearson correlations, Spearman correlations, Lin's concordance correlation coefficient, and Kendall's tau. Sign-concordance rates and heterogeneity statistics were also calculated. Correlation p-values are nominal values from standard correlation tests and assume independent gene-level observations; because genes in a pathway are not fully independent, these analyses are interpreted as profile-level descriptive and comparative statistics rather than as independent mechanistic proof. Influential genes within enriched sets were identified using leave-one-out analyses, in which the change in Stouffer Z-score was measured after each gene was removed from the set.

Statistical thresholds and multiple-testing correction

Primary Global enrichment results were considered significant when the permutation p-value was less than 0.05 and supported by a Wilcoxon p-value less than 0.05. Rows meeting only one of these criteria were labelled suggestive, and tissue-level Wilcoxon-only rows were labelled exploratory. Differential and proximity analyses used false discovery rate correction across gene-set comparisons. A strict genome-wide correction was not applied because the analysis was hypothesis-driven and restricted to a pre-specified collection of ageing-related pathways. Both nominal and false discovery rate-adjusted values were retained for interpretation.

As an additional post-hoc multiple-testing inventory, all p-values generated across enrichment, differential, proximity, concordance, and pairwise disease statistical tests were pooled into a single family-wise Benjamini-Hochberg false discovery rate analysis. This pooled inventory included 4913 p-values and identified 131 tests surviving a global false discovery rate of less than 0.05. All analyses were conducted in Python. 

Comprehensive details of the analysis thresholds, overall significant-hit inventory, multiple-testing transparency, profile-proximity and concordance metrics, differential and pairwise statistical tests, leave-one-out influence findings, MHC/complement sensitivity flags, gene-set overlap checks, and family-wise false discovery rate results are provided in the Appendices.

Data availability and ethics

GWAS summary statistics are publicly available through the PGC data portal. GTEx prediction models and S-PrediXcan software are openly accessible through the MetaXcan resource. Custom Python scripts, full gene-set definitions, input provenance manifests with SHA256 hashes, and processed output CSV files were retained with the analysis package and are available upon reasonable request. No individual-level data were accessed. All analyses complied with the data-use agreements of the source consortia. Because the study used only de-identified aggregate summary statistics, it was treated as exempt from institutional review board oversight.

## Results

TWAS results derived from the PGC-SUD 2020 opioid meta-analysis were examined across three phenotypic contrasts: opioid-exposed versus opioid-unexposed controls, opioid dependence cases versus opioid-unexposed controls, and opioid dependence cases versus opioid-exposed controls [3]. These contrasts were used to distinguish genetic liability for initial opioid exposure from liability for progression to dependence among individuals who had already been exposed. TWAS was performed using S-PrediXcan models trained on GTEx v8 brain tissues, and enrichment was tested across a curated set of ageing-related pathways.

Across the three contrasts, the dominant pattern was one of divergence. Genetic associations with opioid exposure liability often showed the opposite direction from associations with dependence liability conditional on exposure. This divergence appeared across multiple ageing-related pathways and was supported by enrichment statistics, profile-level correlations, and gene-level analyses.

The strongest signal appeared in complement and synaptic-pruning pathways in the exposure contrast (Table 1). The REACTOME COMPLEMENT ALL gene set showed marked negative enrichment in the oex uex analysis, with a Stouffer Z-score of -5.82 and a permutation p-value of 0.0007. This finding was supported by a Wilcoxon signed-rank p-value of 0.011 and survived the pooled family-wise false discovery rate inventory with q=0.009. The regional tissue rows were concordant in direction across brain regions involved in reward and emotional processing, including the anterior cingulate cortex BA24, caudate basal ganglia, amygdala, and hippocampus, although these tissue-specific results are interpreted as exploratory because permutation testing was not performed at the tissue level. Key genes driving this negative signal included C1QA, C1QB, C1QC, and C4A. Additional complement-related genes, including CR1, CD46, CD59, CFB, and CFI, showed similar directional patterns in the influence inventory. These findings indicate that genetic liability for opioid exposure is associated with lower predicted expression of complement components and related synaptic-tagging machinery in brain tissue. Given the role of the classical complement cascade in microglial-mediated synapse elimination and synaptic remodeling, this pattern is consistent with a hypothesis of altered neuroimmune surveillance or synaptic refinement processes that may influence vulnerability to initial opioid exposure or reinforcement [22,23].

**Table 1:** Selected global and regional pathway enrichment results for ageing-related gene sets Negative Stouffer Z indicates lower genetically predicted expression associated with liability; positive Stouffer Z indicates higher genetically predicted expression associated with liability. Tissue-level entries are exploratory because permutation p-values were not computed for those rows. Global family-wise q values are from the pooled 4913-test Benjamini-Hochberg inventory where available. Global: unweighted Stouffer combination of tissue-specific S-PrediXcan Z-scores into one gene-level cross-tissue aggregate before pathway testing. Primary nominal: permutation p<0.05 and Wilcoxon p<0.05. Suggestive: only one of the two primary enrichment tests met p<0.05 or the effect size was large. n: number of genes in set with non-missing TWAS statistics; EXP: opioid-exposed controls; UNX: opioid-unexposed controls; DEP: opioid dependence cases; ACC: anterior cingulate cortex; BG: basal ganglia; GO: Gene Ontology; NA: not available or not computed; TWAS: transcriptome-wide association study

Gene set	Contrast	Tissue	n	Stouffer Z	Perm p	Wilcoxon p	Global family-wise q	Classification
Global cross-tissue enrichment								
REACTOME_COMPLEMENT_ALL	EXP vs. UNX	Global	31	-5.821	0.0007	0.011	0.009	Primary; global FDR-supported
ADULT_ASTRO_ALL	EXP vs. UNX	Global	27	-4.505	0.009	0.030	>0.05	Primary nominal
GOBP_SYNAPSE_PRUNING_ALL	EXP vs. UNX	Global	13	-3.652	0.038	0.057	>0.05	Suggestive
HALLMARK_APOPTOSIS_ALL	EXP vs. UNX	Global	112	-3.587	0.038	0.019	>0.05	Primary nominal
MOOTHA_MITOCHONDRIA_ALL	EXP vs. UNX	Global	318	+3.787	0.037	0.132	>0.05	Suggestive
SAUL_SEN_MAYO_ALL	EXP vs. UNX	Global	75	-3.462	0.047	0.043	>0.05	Primary nominal
REACTOME_ENERGY_DEPENDENT_REGULATION_OF_MTOR_BY_LKB1_AMPK_ALL	DEP vs. EXP	Global	18	+4.118	0.016	0.034	>0.05	Primary nominal
REACTOME_NICOTINATE_METABOLISM_ALL	DEP vs. UNX	Global	24	-3.598	0.039	0.046	>0.05	Primary nominal
ADULT_MICRO_ALL	DEP vs. UNX	Global	14	+3.518	0.048	0.028	>0.05	Primary nominal
Regional tissue-specific enrichment								
REACTOME_COMPLEMENT_ALL	EXP vs. UNX	ACC BA24	15	-3.176	NA	0.005	NA	Exploratory tissue-level
REACTOME_COMPLEMENT_ALL	EXP vs. UNX	Amygdala	15	-2.738	NA	0.012	NA	Exploratory tissue-level
REACTOME_COMPLEMENT_ALL	EXP vs. UNX	Caudate BG	14	-2.952	NA	0.035	NA	Exploratory tissue-level
MOOTHA_MITOCHONDRIA_ALL	EXP vs. UNX	Caudate BG	233	+2.495	NA	0.010	NA	Exploratory tissue-level
HALLMARK_OXIDATIVE_PHOSPHORYLATION_ALL	DEP vs. EXP	Caudate BG	85	+2.340	NA	0.011	NA	Exploratory tissue-level
GOBP_REGULATION_OF_SYNAPTIC_PLASTICITY_ALL	DEP vs. EXP	Hippocampus	50	+2.438	NA	0.014	NA	Exploratory tissue-level

A second major finding was the directional opposition of ageing-pathway profiles between exposure and dependence contrasts. Several gene sets showed inverse correlations when TWAS Z-scores were compared across phenotypes. These included apoptosis-related collections such as HALLMARK APOPTOSIS ALL and REACTOME INTRINSIC PATHWAY FOR APOPTOSIS ALL, phosphoinositide 3-kinase-AKT-mTOR signaling, telomere maintenance pathways, NAD metabolism sets, glutamatergic signaling collections, and monoamine-related sets. For comparisons between oex uex and oud oex, Pearson correlations were consistently negative in the highlighted rows, with many p-values less than 0.001 (Table 2). Spearman rank correlations and Lin's concordance coefficients showed similar patterns. The opposition was not confined to a single tissue but was observed across reward-related regions, such as the nucleus accumbens and caudate, as well as cognitive and emotional regions, including the anterior cingulate cortex and hippocampus. This suggests that the genetic architecture linking ageing-related gene expression to opioid exposure is frequently anti-correlated with the architecture linking the same genes to dependence among exposed individuals.

**Table 2:** Profile correlations of TWAS Z-scores between opioid contrasts for selected ageing-related pathways Selected pathways represent a priori ageing-related pathways with sufficient overlapping genes and prominent profile correlations in the Global cross-tissue analysis. Correlations are computed on gene-level Global TWAS Z-scores. Negative r indicates that genes tending toward positive TWAS Z-scores in one contrast tend toward negative TWAS Z-scores in the other contrast. P-values are nominal values from standard correlation tests; genes are not independent, so these p-values are descriptive. Global FDR support indicates that at least one corresponding profile/proximity test for the row survived the pooled family-wise false discovery rate inventory. n: number of genes overlapping between the two contrasts with non-missing TWAS Z-scores in both; r: Pearson correlation; ρ: Spearman rank correlation; EXP-UNX: opioid-exposed versus opioid-unexposed contrast; DEP-EXP: opioid dependence versus opioid-exposed contrast; DEP-UNX: opioid dependence versus opioid-unexposed contrast; TWAS: transcriptome-wide association study; FDR: false discovery rate

Gene set	Contrasts compared	n	r	p r	ρ	p ρ
Opposing profiles: EXP-UNX vs. DEP-EXP						
Reactome Intrinsic Apoptosis	EXP-UNX vs. DEP-EXP	32	-0.647	<0.001	-0.398	0.024
Hallmark Apoptosis	EXP-UNX vs. DEP-EXP	83	-0.593	<0.001	-0.399	<0.001
Hallmark PI3K-AKT-mTOR	EXP-UNX vs. DEP-EXP	64	-0.569	<0.001	-0.533	<0.001
Monoamines	EXP-UNX vs. DEP-EXP	44	-0.548	<0.001	-0.436	0.003
Reactome Telomere	EXP-UNX vs. DEP-EXP	38	-0.504	0.001	-0.533	<0.001
Senescence, Saul/Mayo	EXP-UNX vs. DEP-EXP	50	-0.477	<0.001	-0.421	0.002
Glutamatergic, Gene Ontology	EXP-UNX vs. DEP-EXP	47	-0.467	<0.001	-0.500	<0.001
Intrinsic Apoptosis, Gene Ontology	EXP-UNX vs. DEP-EXP	181	-0.387	<0.001	-0.306	<0.001
Regulation of Synaptic Plasticity, Gene Ontology	EXP-UNX vs. DEP-EXP	97	-0.386	<0.001	-0.408	<0.001
Hallmark Oxidative Phosphorylation	EXP-UNX vs. DEP-EXP	121	-0.370	<0.001	-0.325	<0.001
Mootha PGC	EXP-UNX vs. DEP-EXP	247	-0.328	<0.001	-0.305	<0.001
Concordant profiles: DEP-EXP vs. DEP-UNX						
Reactome Complement	DEP-EXP vs. DEP-UNX	24	+0.683	<0.001	+0.640	<0.001
Senescence, Saul/Mayo	DEP-EXP vs. DEP-UNX	50	+0.517	<0.001	+0.492	<0.001
Hallmark Oxidative Phosphorylation	DEP-EXP vs. DEP-UNX	121	+0.473	<0.001	+0.449	<0.001
Mootha Mitochondria	DEP-EXP vs. DEP-UNX	261	+0.403	<0.001	+0.356	<0.001

Dependence liability showed its clearest enrichment in energy-sensing and nutrient-responsive pathways. The REACTOME ENERGY DEPENDENT REGULATION OF MTOR BY LKB1 AMPK ALL set was positively enriched in the oud oex contrast, with a Stouffer Z-score of +4.12 and a permutation p-value of 0.016. This dependence-specific enrichment was supported by differential statistics (Table 3) when compared with the oex uex profile, including a Mann-Whitney U p-value of 0.020, a paired Wilcoxon p-value of 0.029, and an absolute rank-biserial correlation of 0.48. LAMTOR5, a Ragulator component, emerged as a particularly influential driver, with strong positive TWAS Z-scores in both oud oex, approximately +4.86, and oud uex, approximately +5.68. Other contributing genes included TSC1, PRKAB1, PRKAG1, MTOR, SLC38A9, LAMTOR3, and LAMTOR4. These genes participate in lysosomal localization and the activation of mTORC1 in response to amino acid and nutrient availability [24,25]. The positive signal in dependence, together with the opposing exposure pattern, suggests that genetic variation influencing mTOR nutrient sensing, autophagy, and protein synthesis pathways may be more relevant to the transition from exposure to dependence than to initial exposure itself.

**Table 3:** Differential statistics comparing enrichment profiles between opioid contrasts for key pathways The primary differential test was MWU with FDR correction; WSR was treated as supportive where gene-wise pairing was possible. Discordant MWU and WSR findings are labelled suggestive. NA indicates that the statistic was not retained in the provided summary output. Δ Mean Z: mean gene-level TWAS Z in the first contrast minus mean gene-level TWAS Z in the second contrast; MWU: Mann-Whitney U test; WSR: paired Wilcoxon signed-rank test; d: Cohen's d, oriented as first contrast minus second contrast where available; RBC: rank-biserial correlation, reoriented so positive values indicate higher ranks in the first contrast and negative values indicate lower ranks in the first contrast; n paired: number of overlapping genes used in paired comparison where available; TWAS: transcriptome-wide association study; FDR: false discovery rate

Gene set	Contrasts compared	n paired	Δ Mean Z	MWU p	WSR p	d	RBC	MWU FDR <0.05	Classification
REACTOME_ENERGY_DEPENDENT_REGULATION_OF_MTOR_BY_LKB1_AMPK_ALL	EXP-UNX vs. DEP-EXP	17	-1.60	0.020	0.029	-0.73	-0.48	No	Primary nominal
REACTOME_COMPLEMENT_ALL	EXP-UNX vs. DEP-EXP	26	-1.17	0.039	0.042	-0.78	-0.35	No	Primary nominal; supported by other global FDR tests
GOBP_INTRINSIC_APOPTOTIC_SIGNALING_PATHWAY_ALL	EXP-UNX vs. DEP-EXP	181	-0.28	0.047	0.047	NA	-0.12	No	Primary nominal
ADULT_ASTRO_ALL	EXP-UNX vs. DEP-EXP	19	-1.21	0.085	0.229	-0.60	-0.34	No	Suggestive effect size
EXP-UNX vs. DEP-UNX comparisons									
ADULT_MICRO_ALL	EXP-UNX vs. DEP-UNX	14	-1.33	0.038	0.054	-0.89	-0.51	No	Suggestive; MWU only
WP_NAD_METABOLISM_SIRTUINS_AND_AGING_ALL	EXP-UNX vs. DEP-UNX	10	+1.30	0.141	0.106	+0.73	+0.40	No	Suggestive effect size
SAUL_SEN_MAYO_ALL	EXP-UNX vs. DEP-UNX	53	-0.52	0.185	0.047	-0.23	-0.15	No	Suggestive; paired test only

Mitochondrial function and oxidative phosphorylation also showed dependence-related signals. MOOTHA MITOCHONDRIA ALL and HALLMARK OXIDATIVE PHOSPHORYLATION ALL were positively enriched in the oud oex and oud uex contrasts, with especially strong contributions from the caudate basal ganglia. DLD, which encodes dihydrolipoamide dehydrogenase, showed exceptionally high positive TWAS Z-scores of approximately +7.0 in oud oex and +7.6 in oud uex. DLD participates in multiple mitochondrial dehydrogenase complexes, including pyruvate dehydrogenase and alpha-ketoglutarate dehydrogenase complexes, placing it at an important metabolic junction between glycolysis, the tricarboxylic acid cycle, and reduced NAD production [26,27]. Other contributors included respiratory chain genes, such as NDUFS1, NDUFC2, COX5A, and CYC1, as well as tricarboxylic acid and dehydrogenase-related genes, including OGDH, PDHB, and DLST. The consistent positive direction in dependence contrasts, compared with more modest or mixed findings in the exposure contrast, supports a hypothesis in which dependence liability involves altered mitochondrial energetic handling or compensatory responses to chronic opioid-related metabolic stress.

NAD metabolism and sirtuin-related pathways showed a more complex pattern. The REACTOME NICOTINATE METABOLISM ALL set was negatively enriched in the oud uex contrast, with a Stouffer Z-score of -3.60 and a permutation p-value of 0.039. At the same time, WP NAD METABOLISM SIRTUINS AND AGING ALL showed positive signals in dependence contrasts. Gene-level analysis revealed a sign-flip for NAMPT, the rate-limiting enzyme in the NAD salvage pathway. NAMPT showed a positive TWAS Z-score in oex uex but a negative Z-score of approximately -2.85 in oud oex. In contrast, SIRT6 showed positive associations in both oud oex, approximately +3.92, and oud uex, approximately +2.18. NT5E, which encodes CD73, an ecto-5-prime-nucleotidase involved in extracellular adenosine generation, was strongly positive in oud oex, with a Z-score of approximately +3.98. These results suggest that dependence liability may involve a shift away from NAMPT-driven NAD salvage and toward SIRT6-related DNA repair, chromatin regulation, and adenosine signaling. This pattern is consistent with known roles of NAD metabolism and sirtuins in stress responses and ageing [7,28].

Influential-gene analyses identified several large directional changes across enriched sets. CASP7 contributed positively to the exposure contrast but negatively and substantially to the dependence contrast, producing one of the largest gene-level directional shifts observed (Table 4). TERF2, a shelterin-complex component, showed an exposure-negative and dependence-positive pattern. Among presynaptic vesicle and release-related genes, STX1A was consistently negative across dependence contrasts, whereas ADCY6 showed opposing directions between exposure, where it was positive, and oud oex, where it was negative. These gene-level patterns reinforced the broader pathway-level divergence and highlighted specific molecular nodes that may distinguish exposure liability from dependence liability.

**Table 4:** TWAS Z-scores for selected influential genes across opioid contrasts Z-scores are S-PrediXcan gene-level association statistics after Global cross-tissue Stouffer aggregation. Genes were prioritized using leave-one-out influence on pathway enrichment; leave-one-out was not used to create the gene-level Z-scores themselves. NA indicates that the value was not available in the provided summary output and should not be interpreted as zero association. ‡ denotes a directional sign-flip between EXP-UNX and DEP-EXP, defined as opposite signs of the available Global TWAS Z-scores. EXP-UNX: opioid-exposed versus opioid-unexposed contrast; DEP-EXP: opioid dependence versus opioid-exposed contrast; DEP-UNX: opioid dependence versus opioid-unexposed contrast; TWAS: transcriptome-wide association study

Gene	Primary pathway context	Z EXP-UNX	Z DEP-EXP	Z DEP-UNX
C1QC	Complement/synapse pruning	-3.69	+0.12	-
C1QA	Complement/synapse pruning	-3.46	-	-
GRM3	Glutamatergic/astrocyte	-2.73	-	-4.31
CASP7^‡^	Apoptosis/oxidative phosphorylation	+7.05	-5.81	-
TERF2^‡^	Telomere/senescence	-2.99	+2.93	-
NAMPT^‡^	Nicotinamide adenine dinucleotide salvage	+1.78	-2.85	-
SIRT6^‡^	Nicotinamide adenine dinucleotide/sirtuin/senescence	-1.30	+3.92	+2.18
NT5E^‡^	Nicotinate metabolism/adenosine	-1.65	+3.98	-
ADCY6^‡^	Cyclic adenosine monophosphate signaling/glucagon-like peptide 1	+5.33	-1.93	+3.83
DLD	Oxidative phosphorylation/tricarboxylic acid cycle	-	+7.01	+7.62
LAMTOR5	Mechanistic target of rapamycin/Ragulator complex	+1.48	+4.86	+5.68
STX1A	Presynaptic vesicle release	-	-2.67	-3.39

Astrocyte and glutamatergic pathways provided additional support for the core pattern. The ADULT ASTRO ALL collection showed negative enrichment in oex uex, with a Stouffer Z-score of -4.51 and a permutation p-value of 0.009. Notable contributing genes included GRM3, ATP1A2, and SLC1A3. These genes are involved in astrocytic glutamate uptake and metabotropic glutamate receptor signaling, processes linked to glutamate homeostasis and addiction-related relapse biology [29,30]. Their negative association with exposure liability complements the complement and pruning findings, suggesting coordinated glial and neuroimmune alterations at the exposure stage.

Post-hoc robustness and sensitivity analyses were then performed on the full pipeline output. The pooled statistical inventory included 4913 p-values across enrichment, differential, proximity, pairwise, and concordance tests; 131 survived global Benjamini-Hochberg false discovery rate less than 0.05. These globally corrected findings were concentrated in proximity and concordance analyses, with 63 proximity tests and 66 pairwise or concordance tests surviving correction, together with two enrichment entries. The automated opposing-profile screen identified an opposing exposure-versus-dependence pattern in 100 of 102 evaluable pathway-profile entries. Leave-one-out influence analysis flagged 5074 influential gene-disease entries before deduplication, with recurrent high-influence genes including MYH7B, SIRT6, CAMKK2, SIRT5, STX1A, HTT, CRYL1, and VAMP2. MHC/complement sensitivity flagging identified 10 immune or complement-containing gene sets, including REACTOME COMPLEMENT ALL, HLA_COMPLEX_ALL, GOBP_SYNAPSE_PRUNING_ALL, and ADULT_MICRO_ALL. Gene-set overlap analysis showed that one Reactome senescence pair was fully redundant, with Jaccard overlap of 1.000, and that 45 gene sets retained at least three unique non-shared genes. Tissue-specific decomposition found no significant Kruskal-Wallis heterogeneity across 149 gene-set-by-contrast combinations at p<0.05.

Taken together, these results indicate that genetic liability for opioid exposure and genetic liability for dependence among exposed individuals are associated with distinct and often opposing patterns of ageing-related gene expression in brain tissue. Exposure liability was most strongly linked to reduced complement-mediated synaptic pruning and glial support, whereas dependence liability was characterized by positive enrichment of AMPK-mTOR nutrient sensing, mitochondrial oxidative phosphorylation, selected NAD/sirtuin pathways, presynaptic adaptation, and DNA repair-related processes.

## Discussion

**Figure 1:**
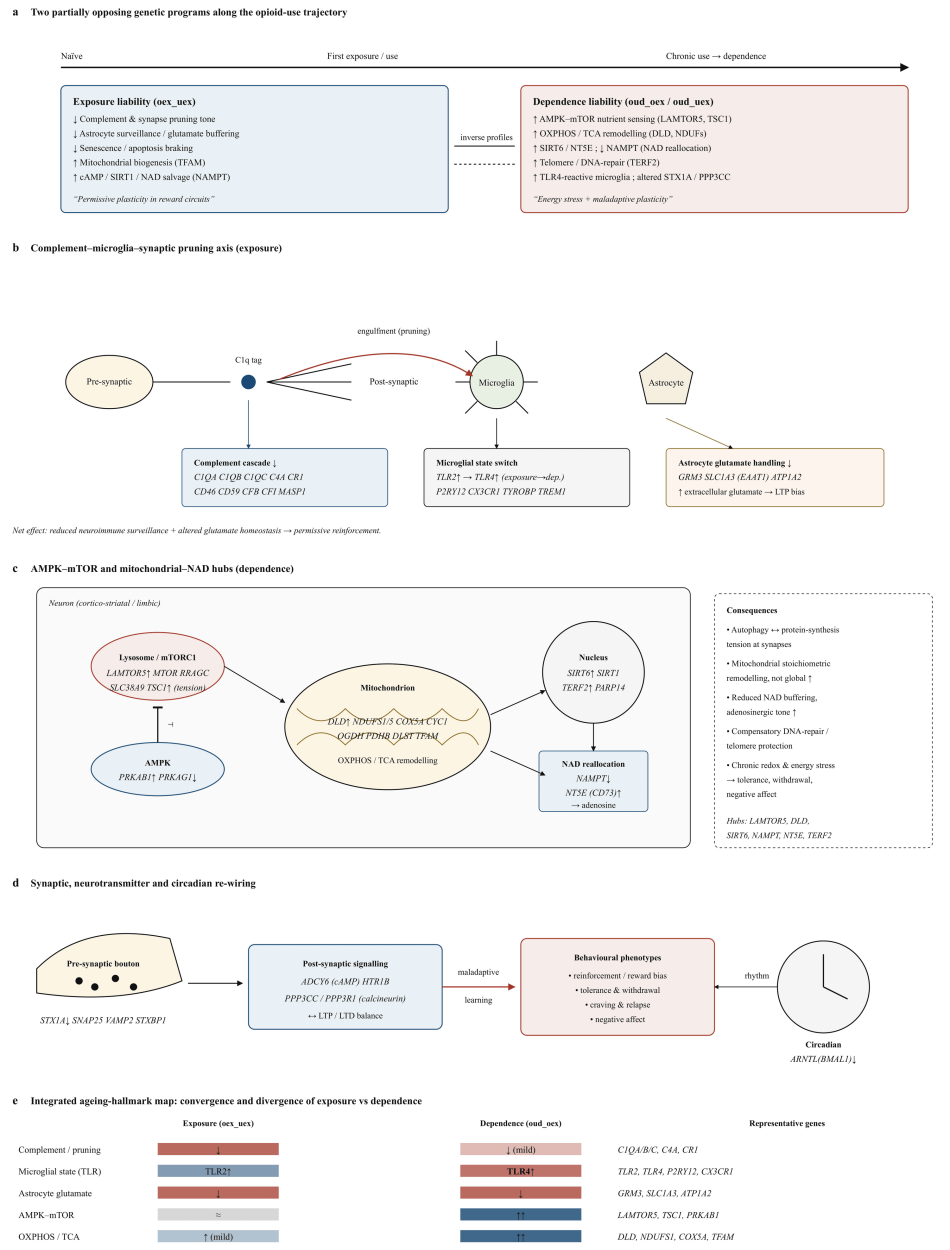
Gene-level mechanistic model of opioid use disorder liability across ageing hallmarks (a) Two partially opposing genetic programs map onto the opioid use trajectory: an exposure program marked by reduced neuroimmune pruning tone and permissive synaptic plasticity and a dependence program dominated by nutrient sensing, mitochondrial, and NAD reallocation under chronic drug stress. Profiles of many ageing pathways are inversely correlated across the 2 contrasts, indicating that exposure and dependence are not a simple transcriptomic continuum. (b) Complement-microglia-synaptic-pruning axis: lower genetically predicted expression of C1q and C4A components, combined with altered astrocytic glutamate handling (GRM3, SLC1A3, ATP1A2) and a shift from TLR2- to TLR4-dominant microglial states, plausibly weakens neuroimmune surveillance of cortico-striatal and limbic synapses. (c) AMPK-mTOR, mitochondrial, and NAD hubs define the dependence program. Elevated lysosomal Ragulator signalling (LAMTOR5) with co-elevated TSC1 creates a regulatorily tense mTORC1 state; mitochondrial stoichiometric remodelling (DLD, respiratory-chain subunits, TFAM) coexists with a shift from NAMPT/SIRT1-dominant NAD salvage toward SIRT6/NT5E-driven reallocation and adenosinergic tone. (d) Synaptic-neurotransmitter-circadian rewiring couples reduced presynaptic release machinery (STX1A) and altered postsynaptic cAMP/serotonergic/calcineurin signalling (ADCY6, HTR1B, PPP3CC) to behavioral endpoints of tolerance, withdrawal, and relapse, under circadian control (ARNTL). (e) Integrated hallmark map summarizing directional concordance and divergence between exposure and dependence across 9 ageing-related domains. Blue and red denote higher and lower genetically predicted expression associated with risk; representative priority genes for colocalization and iPSC-based validation are listed on the right. All directions reflect statistical associations between genetically predicted expression and trait liability and require functional validation. Image created by Ngo Cheung using PowerPoint

This TWAS analysis of PGC-SUD 2020 opioid summary statistics identified a stage-specific pattern in ageing-related brain gene expression. Genetic liability for opioid exposure was most strongly associated with reduced predicted expression of complement components and synaptic-pruning machinery, along with negative signals in astrocyte and selected senescence-related gene sets. In contrast, liability for dependence among exposed individuals was marked by positive enrichment of AMPK-mTOR nutrient-sensing pathways, mitochondrial oxidative phosphorylation, and selected NAD/sirtuin signaling, together with presynaptic and DNA repair adaptations (Figure 1).

The most striking feature was that many ageing-related pathways showed opposing directional profiles between exposure and dependence contrasts. This suggests that these biological profiles are not merely more active or less active along a single continuum of opioid involvement, but may instead be associated differently across stages.

The negative complement signal in the exposure contrast is consistent with evidence that the classical complement cascade, including C1q and C4-related mechanisms, contributes to synaptic tagging and microglial-mediated synapse elimination during development and plasticity [22,23]. Reduced predicted expression of these components may indicate altered synaptic refinement in cortico-striatal and limbic circuits. Such altered refinement could plausibly affect initial opioid reinforcement or neuroimmune feedback systems that might otherwise constrain escalation. The broader involvement of innate immune and glial pathways also fits with the literature suggesting that neuroimmune signaling contributes to opioid tolerance, reward, and dependence [12]. Because several complement genes lie in or near genomic regions with complex linkage disequilibrium, particularly the MHC region, these findings should be treated as strong TWAS association signals rather than proof of causal complement-gene effects. Dedicated colocalization and fine-mapping will be required before causal interpretation.

The dependence-specific enrichment of AMPK-mTOR and mitochondrial pathways is consistent with the idea that chronic opioid exposure may impose metabolic stress and engage energy-sensing profiles. mTORC1 signaling, regulated in part by the Ragulator complex containing LAMTOR5, integrates nutrient availability with autophagy, protein synthesis, and synaptic plasticity [24,25]. The strong LAMTOR5 contribution in the dependence contrast suggests that genetic variation influencing lysosomal mTOR recruitment may become particularly relevant after opioid exposure, when neuroadaptations begin to consolidate. The strong DLD signal and broader enrichment of oxidative phosphorylation and tricarboxylic acid-related genes similarly point toward mitochondrial energetic pathways as a feature of dependence liability. Opioid and psychostimulant neurotoxicity have been linked to cellular and mitochondrial mechanisms, supporting the interpretation that repeated exposure may create or reveal vulnerability in energy-handling systems [11].

The NAD/sirtuin pattern suggests a possible reallocation of cellular stress-response systems across opioid stages. NAMPT showed a positive association with exposure but a negative association with dependence among exposed individuals, whereas SIRT6 and NT5E showed positive dependence-related associations. NAD metabolism and sirtuin activity are central to energy metabolism, DNA repair, chromatin regulation, senescence, and immune-cell function during ageing [7,28]. In this context, the dependence pattern may reflect a shift from NAD salvage toward stress-adaptive pathways involving SIRT6-linked chromatin or DNA repair mechanisms and NT5E-related adenosine signaling. This interpretation remains hypothetical, but it provides a biologically coherent link between ageing-related stress biology and dependence liability.

Gene-level findings strengthened the staged interpretation. CASP7 showed one of the clearest sign-flips, contributing positively to exposure but negatively to dependence. TERF2 showed an exposure-negative and dependence-positive pattern, suggesting that telomere-maintenance or shelterin-related mechanisms may differ across opioid stages. STX1A was consistently negative across dependence contrasts, while ADCY6 showed an exposure-positive and dependence-negative pattern. These genes point toward apoptosis-related signaling, telomere protection, presynaptic vesicle release, and cyclic adenosine monophosphate-related association profiles as candidate mechanisms that may separate exposure liability from dependence liability. Together with pathway-level results, these gene-level patterns suggest that exposure is weighted toward neuroimmune and glial alterations, whereas dependence is weighted toward metabolic, mitochondrial, and synaptic association profiles.

The post-hoc robustness package strengthened the statistical description of the main finding while also clarifying its limits. The exposure-versus-dependence opposition was observed in 100 of 102 evaluable pathway-profile entries in the automated inventory, and 131 of 4913 pooled tests survived a single global false discovery rate threshold. Tissue-specific decomposition did not identify significant heterogeneity across 149 gene-set-by-contrast combinations, suggesting that the Global Stouffer signals were not simply an artefact of one highly discordant tissue. At the same time, the sensitivity inventory identified important caveats: one Reactome senescence pair was redundant, MHC/complement-containing sets require linkage disequilibrium-sensitive validation, and leave-one-out analyses showed that some small gene sets are highly sensitive to individual genes. These checks support the general opposing-profile observation but also justify treating individual pathway and gene claims as hypothesis-generating.

Several strengths support the interpretation of these findings. The use of three opioid contrasts allowed exposure liability to be separated from dependence liability, addressing a common limitation of traditional OUD case-control designs. The analysis combined enrichment testing, differential testing, profile correlations, concordance metrics, and influential-gene analysis, reducing reliance on a single statistical measure. Brain-specific TWAS models and a focused collection of ageing-related gene sets increased biological relevance while limiting the search space. The gene-level results also helped identify plausible mechanistic nodes rather than leaving the interpretation at the level of broad pathway labels. The additional pooled false discovery rate inventory, tissue decomposition, gene-set overlap analysis, MHC/complement flagging, and leave-one-out influence analysis further increased transparency and made it possible to distinguish primary, suggestive, and exploratory findings.

The findings should nevertheless be interpreted cautiously. TWAS associations reflect genetically predicted expression, not measured brain expression, and they can be affected by linkage disequilibrium, pleiotropy, and imperfect prediction models. They do not establish causal direction or confirm the specific tissue or cell type in which a gene acts. The MHC region, which includes complement genes such as C4A, is especially difficult to interpret because of complex linkage structure and should be examined with dedicated fine-mapping in future work. Bulk brain tissue models cannot fully resolve cell type-specific contributions, although the complement, innate immune, astrocytic, and glutamatergic signals are consistent with plausible microglial and astrocytic involvement. Gene-set overlap and correlated expression among ageing-related pathways may also inflate apparent convergence. The Global Stouffer aggregation did not explicitly model cross-tissue correlation and may inflate signal strength when tissue-specific Z-scores are correlated. Tissue-level analyses were therefore used as exploratory checks rather than independent confirmation. Finally, because the source GWAS data are cross-sectional, the temporal ordering of exposure and dependence processes cannot be directly inferred.

Despite these limitations, the results suggest several translational directions. One immediate opportunity is the development of stage-specific genetic risk modules. An exposure-pruning module built around complement and astrocyte genes could be tested for association with age at first opioid use, subjective reinforcing effects, or transition from medical exposure to misuse. A dependence-metabolic module centered on LAMTOR5, DLD, SIRT6, and NAD-related genes could be tested for the prediction of DSM-defined dependence, withdrawal severity, or relapse risk following detoxification. These modular approaches may capture more specific biology than single polygenic scores that combine heterogeneous liability processes.

The findings also generate biomarker hypotheses. Complement protein levels, NAD and reduced NAD balance, adenosine metabolites, and mitochondrial markers such as lactate, pyruvate, or tricarboxylic acid intermediates could be examined in longitudinal cohorts of opioid-exposed individuals to determine whether they track exposure liability, progression to dependence, or treatment response. Imaging measures related to striatal or anterior cingulate glutamate could also be evaluated alongside astrocyte and glutamate-related TWAS modules, given the role of glutamate homeostasis in addiction models [29,30]. Peripheral and imaging biomarkers would be more practical than direct brain expression measurements and could eventually support stratified clinical studies.

Therapeutic implications remain preliminary. Glutamate homeostasis modulators, especially approaches that normalize astrocytic glutamate handling or metabotropic glutamate signaling, could be prioritized for individuals with high astrocyte or glutamate-related genetic risk. Interventions that support mitochondrial resilience or NAD availability may be worth examining as adjunctive strategies for withdrawal or protracted abstinence symptoms, particularly if biomarkers indicate energetic or redox stress. Autophagy and mTOR-related pathways may also be relevant to dependence-related plasticity, though safety, timing, and target specificity would require careful study. Adenosine signaling pathways, suggested by NT5E, offer another potential direction because of their relevance to sleep, motivation, and neuroimmune regulation. These ideas should not be taken as immediate clinical recommendations; they require validation in cellular systems, animal models, and carefully designed human studies.

Future work should prioritize colocalization and fine-mapping of key driver genes, including LAMTOR5, DLD, SIRT6, NAMPT, NT5E, C1QA, C1QB, C1QC, C4A, GRM3, STX1A, ADCY6, and CASP7. Such analyses will help distinguish causal expression effects from signals produced by linkage disequilibrium. Longitudinal cohorts following opioid-naïve or recently exposed individuals with genetic, transcriptomic, metabolomic, and clinical data could test whether the identified modules prospectively predict exposure or progression outcomes. Induced pluripotent stem cell-derived microglia, astrocytes, and striatal neurons exposed to repeated opioid and withdrawal paradigms may provide useful functional systems for testing the implicated genes. Integration of TWAS modules with epigenetic age, telomere length, metabolomics, and neuroimaging in OUD cohorts would further clarify how addiction and ageing-related biology intersect.

Overall, the results support an emerging view that substance use disorders intersect with fundamental ageing processes at the genetic and transcriptomic level. By showing that exposure and dependence liabilities engage overlapping but directionally distinct ageing-related profiles, this study highlights the value of phenotype decomposition in psychiatric genetics. The pattern of reduced neuroimmune pruning in exposure liability and metabolic-synaptic association in dependence liability offers a biologically grounded framework for future mechanistic and translational research in OUD.

## Conclusions

This TWAS analysis of the PGC-SUD 2020 opioid meta-analysis suggests that genetic liability for opioid exposure and genetic liability for dependence among exposed individuals are associated with partially opposing ageing-related gene expression profiles in brain tissue. Exposure liability was most robustly linked to reduced complement-mediated synaptic pruning and glial support. Dependence liability was characterized by increased AMPK-mTOR nutrient sensing, mitochondrial oxidative phosphorylation, especially involving DLD, and selected NAD/sirtuin pathways, together with presynaptic and DNA repair-associated signals. These stage-specific signatures were supported by convergent statistical evidence and align with established mechanisms in neuroimmune signaling, glutamate homeostasis, mitochondrial function, and synaptic plasticity. The findings were further supported by an extensive post-hoc sensitivity inventory, including 131 of 4913 pooled tests surviving strict global false discovery rate correction, an opposing-profile criterion met by 100 of 102 evaluable pathway-profile entries, and no significant tissue heterogeneity across 149 tissue decomposition tests. At the same time, MHC/complement linkage disequilibrium, gene-set redundancy, and single-gene influence remain important caveats requiring future validation.

The findings have implications for risk stratification, biomarker development, and the design of stage-matched prevention and treatment strategies. Future validation through colocalization, longitudinal cohorts, and functional models will be essential before these associations can be translated into actionable clinical insights. Recognizing that exposure and dependence may involve biologically distinct ageing-related profiles provides a more nuanced foundation for precision approaches to OUD prevention and treatment.

## Appendices

**Table 5:** Analysis thresholds and overall significant-hit inventory MWU: Mann-Whitney U test; FDR: false discovery rate; CCC: concordance correlation coefficient; LOO: leave-one-out

Domain	Criterion or count	Value	Interpretive note
Raw p-value threshold	p	<0.05	Nominal threshold used for screening
FDR/global FDR threshold	q	<0.05	Used where FDR-adjusted values were available
Stouffer Z threshold	|Z|	>1.96	Directional enrichment magnitude threshold
Cohen's d threshold	|d|	>0.5	Effect-size threshold for pairwise contrasts
Lin's CCC threshold	|CCC|	>0.5	Concordance/discordance threshold
Sign-concordance threshold	Rate	>0.7	Used for paired sign agreement
Kendall tau threshold	|τ|	>0.3	Rank-concordance threshold
Pearson r threshold	|r|	>0.3	Profile-proximity threshold
Rank-biserial threshold	|RBC|	>0.3	Differential distribution threshold
Per-disease enrichment	Significant findings	88	Includes Global and tissue-level entries
Differential tests	Significant pairs	17	Kruskal-Wallis plus pairwise framework
Profile proximity	Significant pairs	69	Pearson/Spearman/cosine profile similarity
Pairwise disease statistical tests	Significant comparisons	23	Includes MWU, Welch, Brunner-Munzel, paired tests, and permutation tests
Concordance metrics	Significant pairs	51	Sign concordance, CCC, and Kendall tau
LOO influence	Influential entries	2537	Only sign-flip entries are tabulated below
Family-wise FDR	FDR-significant entries	121	Pooled across in-run p-values in sig_summary
Grand total	Significant hits	2906	Total reported in sig_summary

**Table 6:** Multiple-testing transparency and threshold robustness Signals persisting under the strict FDR + |Stouffer Z|>2.5 regime are the most defensible. Differences between nominal and strict columns identify near-threshold or exploratory findings. FDR: false discovery rate

Section	Nominal p<0.01	FDR<0.05 + |Z|>2.5	Full FDR<0.05
Concordance	20	20	51
Differential	12	12	12
Enrichment	20	20	88
Family-wise FDR	20	20	121
Influential genes	20	20	2537
Pairwise stat tests	17	16	16
Proximity	20	20	69
Grand total	129	128	2894

**Table 7:** Global enrichment results and selected tissue-level decomposition FDR: false discovery rate

Gene set	Contrast	Tissue	n	Stouffer Z	Permutation p	Wilcoxon p	Classification
REACTOME_COMPLEMENT_ALL	oex_uex	Global	31	-5.821	0.0007	0.01108	Primary; global FDR-supported
ADULT_ASTRO_ALL	oex_uex	Global	27	-4.505	0.009299	0.02991	Primary nominal
REACTOME_ENERGY_DEPENDENT_REGULATION_OF_MTOR_BY_LKB1_AMPK_ALL	oud_oex	Global	18	4.118	0.016	0.03423	Primary nominal
MOOTHA_MITOCHONDRIA_ALL	oex_uex	Global	318	3.787	0.0365	0.1318	Suggestive; permutation/Stouffer only
HALLMARK_APOPTOSIS_ALL	oex_uex	Global	112	-3.587	0.0377	0.01903	Primary nominal
GOBP_SYNAPSE_PRUNING_ALL	oex_uex	Global	13	-3.652	0.0382	0.05737	Suggestive; Wilcoxon near-threshold
REACTOME_NICOTINATE_METABOLISM_ALL	oud_uex	Global	24	-3.598	0.0388	0.04568	Primary nominal
SAUL_SEN_MAYO_ALL	oex_uex	Global	75	-3.462	0.0468	0.04259	Primary nominal
ADULT_MICRO_ALL	oud_uex	Global	14	3.518	0.048	0.02771	Primary nominal
Selected tissue-level entries; permutation p-values are not computed and rows are exploratory							
REACTOME_COMPLEMENT_ALL	oex_uex	Brain_Anterior_cingulate_cortex_BA24	15	-3.176	NA	0.005371	Exploratory tissue-level
REACTOME_COMPLEMENT_ALL	oex_uex	Brain_Amygdala	15	-2.738	NA	0.01245	Exploratory tissue-level
REACTOME_COMPLEMENT_ALL	oex_uex	Brain_Caudate_basal_ganglia	14	-2.952	NA	0.03528	Exploratory tissue-level
REACTOME_COMPLEMENT_ALL	oex_uex	Brain_Hippocampus	16	-3.112	NA	0.05768	Exploratory tissue-level; Z-supported
MOOTHA_MITOCHONDRIA_ALL	oex_uex	Brain_Caudate_basal_ganglia	233	2.495	NA	0.01048	Exploratory tissue-level
HALLMARK_OXIDATIVE_PHOSPHORYLATION_ALL	oud_oex	Brain_Caudate_basal_ganglia	85	2.340	NA	0.01118	Exploratory tissue-level
GOBP_REGULATION_OF_SYNAPTIC_PLASTICITY_ALL	oud_oex	Brain_Hippocampus	50	2.438	NA	0.01383	Exploratory tissue-level
REACTOME_NICOTINATE_METABOLISM_ALL	oud_uex	Brain_Nucleus_accumbens_basal_ganglia	17	-2.375	NA	0.005569	Exploratory tissue-level
GOBP_REGULATION_OF_CELLULAR_SENESCENCE_ALL	oud_uex	Brain_Anterior_cingulate_cortex_BA24	13	-2.453	NA	0.01343	Exploratory tissue-level
ADULT_MICRO_ALL	oud_uex	Brain_Amygdala	7	2.757	NA	0.04688	Exploratory tissue-level

**Table 8:** Differential tests between opioid contrasts MWU: Mann-Whitney U test; RBC: rank-biserial correlation; FDR: false discovery rate

Gene set	Comparison	Mean Z diff	MWU p	MWU FDR	Paired W p	RBC	Interpretation
REACTOME_ENERGY_DEPENDENT_REGULATION_OF_MTOR_BY_LKB1_AMPK_ALL	oex_uex vs oud_oex	-1.603	0.02046	0.06137	0.02899	0.484	Nominal; medium RBC
ADULT_MICRO_ALL	oex_uex vs oud_uex	-1.333	0.03762	0.1129	0.05371	0.507	Nominal; medium RBC
REACTOME_COMPLEMENT_ALL	oex_uex vs oud_oex	-1.172	0.03921	0.1176	0.04157	0.349	Nominal; medium RBC
GOBP_INTRINSIC_APOPTOTIC_SIGNALING_PATHWAY_ALL	oex_uex vs oud_oex	-0.279	0.04671	0.1401	0.04662	0.121	Nominal; small RBC
GOBP_REGULATION_OF_CELLULAR_SENESCENCE_ALL	oex_uex vs oud_oex	-0.956	0.07028	0.2109	0.2101	0.346	Effect-size supported
ADULT_ASTRO_ALL	oex_uex vs oud_oex	-1.212	0.08465	0.254	0.2288	0.340	Effect-size supported
WP_NAD_METABOLISM_SIRTUINS_AND_AGING_ALL	oex_uex vs oud_uex	1.299	0.1405	0.243	0.1055	-0.400	Effect-size supported
WP_NAD_METABOLISM_SIRTUINS_AND_AGING_ALL	oex_uex vs oud_oex	1.105	0.162	0.243	0.1934	-0.380	Effect-size supported
REACTOME_SIRT1_NEGATIVELY_REGULATES_RRNA_EXPRESSION_ALL	oex_uex vs oud_oex	1.096	0.1797	0.539	0.2188	-0.500	Effect-size supported
SAUL_SEN_MAYO_ALL	oex_uex vs oud_uex	-0.518	0.1845	0.2767	0.04719	0.154	Paired W nominal
GOBP_NAD_TRANSPORT_ALL	oex_uex vs oud_uex	1.142	0.2	0.6	0.5	-0.778	Large RBC; very small n
ADULT_MICRO_ALL	oud_oex vs oud_uex	-0.663	0.2481	0.2481	0.02441	0.285	Paired W nominal
GOBP_POSITIVE_REGULATION_OF_MICROGLIAL_ACTIVATION	oex_uex vs oud_oex	0.603	0.2893	0.5658	0.4961	-0.309	Effect-size supported
GOBP_GLUTAMATERGIC_ALL	oex_uex vs oud_uex	0.397	0.3055	0.4583	0.04616	-0.123	Paired W nominal
GOBP_CAMKK_AMPK_SIGNALING_CASCADE_ALL	oud_oex vs oud_uex	-0.590	0.3829	0.6836	0.5781	0.306	Effect-size supported
REACTOME_SIRT1_NEGATIVELY_REGULATES_RRNA_EXPRESSION_ALL	oex_uex vs oud_uex	1.218	0.4848	0.7273	0.03125	-0.278	Paired W nominal
GOBP_NAD_TRANSPORT_ALL	oex_uex vs oud_oex	1.390	0.7	1	0.75	-0.333	Effect-size supported; very small n

**Table 9:** Profile-proximity results: strongest opposing and concordant pathway profiles

Gene set	Comparison	n	Pearson r	p	Spearman ρ	p	Profile direction
GOBP_POSITIVE_REGULATION_OF_MICROGLIAL_ACTIVATION	oex_uex vs oud_oex	9	-0.938	0.00018	-0.900	0.000943	Opposing
REACTOME_SYNTHESIS_SECRETION_AND_INACTIVATION_OF_GLP_1_ALL	oex_uex vs oud_oex	8	-0.898	0.002459	-0.571	0.139	Opposing
REACTOME_GLUCAGON_LIKE_PEPTIDE_1_GLP1_REGULATES_INSULIN_SECRETION_ALL	oex_uex vs oud_oex	21	-0.726	0.000196	-0.617	0.002895	Opposing
GOBP_SYNAPSE_PRUNING_ALL	oex_uex vs oud_oex	10	-0.729	0.01678	-0.782	0.007547	Opposing
REACTOME_INTRINSIC_PATHWAY_FOR_APOPTOSIS_ALL	oex_uex vs oud_oex	32	-0.647	0.000064	-0.398	0.02418	Opposing
WP_NAD_METABOLISM_ALL	oex_uex vs oud_oex	11	-0.649	0.03076	-0.700	0.01647	Opposing
HLA_COMPLEX_ALL	oex_uex vs oud_oex	16	-0.621	0.01026	-0.523	0.03741	Opposing
MONOAMINES_ALL	oex_uex vs oud_oex	44	-0.548	0.000116	-0.436	0.003097	Opposing
HALLMARK_APOPTOSIS_ALL	oex_uex vs oud_oex	83	-0.593	0	-0.399	0.000189	Opposing
HALLMARK_PI3K_AKT_MTOR_SIGNALING_ALL	oex_uex vs oud_oex	64	-0.569	0.000001	-0.533	0.000006	Opposing
REACTOME_TELOMERE_ALL	oex_uex vs oud_oex	38	-0.504	0.001244	-0.533	0.000578	Opposing
SAUL_SEN_MAYO_ALL	oex_uex vs oud_oex	50	-0.477	0.000461	-0.421	0.00232	Opposing
GOBP_GLUTAMATERGIC_ALL	oex_uex vs oud_oex	47	-0.467	0.000948	-0.500	0.000348	Opposing
HALLMARK_OXIDATIVE_PHOSPHORYLATION_ALL	oex_uex vs oud_oex	121	-0.370	0.00003	-0.325	0.000276	Opposing
MOOTHA_PGC_ALL	oex_uex vs oud_oex	247	-0.328	0	-0.305	0.000001	Opposing
Selected concordant dependence-related profiles							
REACTOME_COMPLEMENT_ALL	oud_oex vs oud_uex	24	0.683	0.000234	0.640	0.000757	Concordant
HALLMARK_OXIDATIVE_PHOSPHORYLATION_ALL	oud_oex vs oud_uex	121	0.473	0	0.449	0	Concordant
MOOTHA_MITOCHONDRIA_ALL	oud_oex vs oud_uex	261	0.403	0	0.356	0	Concordant
MOOTHA_PGC_ALL	oud_oex vs oud_uex	247	0.473	0	0.421	0	Concordant
SAUL_SEN_MAYO_ALL	oud_oex vs oud_uex	50	0.517	0.000122	0.492	0.000287	Concordant

**Table 10:** Concordance metrics for key opposing and dependence-concordant profiles CCC: concordance correlation coefficient; FDR: false discovery rate

Gene set	Comparison	n paired	Sign rate	Sign p	CCC	Kendall τ	τ p/FDR
HALLMARK_PI3K_AKT_MTOR_SIGNALING_ALL	oex_uex vs oud_oex	65	0.250	0.000077	-0.563	-0.385	0.0001125
HALLMARK_APOPTOSIS_ALL	oex_uex vs oud_oex	88	0.420	0.1654	-0.565	-0.286	0.000082
SAUL_SEN_MAYO_ALL	oex_uex vs oud_oex	55	0.400	0.177	-0.496	-0.351	0.001491
GOBP_GLUTAMATERGIC_ALL	oex_uex vs oud_oex	51	0.320	0.01535	-0.450	-0.351	0.002122
REACTOME_TELOMERE_ALL	oex_uex vs oud_oex	40	0.316	0.03355	-0.504	-0.386	0.003236
GOBP_POSITIVE_REGULATION_OF_MICROGLIAL_ACTIVATION	oex_uex vs oud_oex	11	0.273	0.2266	-0.802	-0.673	0.01479
REACTOME_GLUCAGON_LIKE_PEPTIDE_1_GLP1_REGULATES_INSULIN_SECRETION_ALL	oex_uex vs oud_oex	21	0.333	0.1892	-0.660	-0.457	0.01479
MONOAMINES_ALL	oex_uex vs oud_oex	49	0.286	0.003802	-0.506	-0.286	0.003776
WP_NAD_METABOLISM_ALL	oex_uex vs oud_oex	11	0.182	0.06543	-0.576	-0.491	0.04053
Dependence-concordant examples							
HALLMARK_OXIDATIVE_PHOSPHORYLATION_ALL	oud_oex vs oud_uex	123	0.667	0.00033	0.471	0.318	0
GOBP_INTRINSIC_APOPTOTIC_SIGNALING_PATHWAY_ALL	oud_oex vs oud_uex	182	0.639	0.000239	0.441	0.311	0
HALLMARK_APOPTOSIS_ALL	oud_oex vs oud_uex	85	0.631	0.02138	0.423	0.339	0.00008571
REACTOME_COMPLEMENT_ALL	oud_oex vs oud_uex	24	0.652	0.21	0.613	0.485	0.004019
ADULT_MICRO_ALL	oud_oex vs oud_uex	12	0.909	0.01172	0.614	0.576	0.03284

**Table 11:** Pairwise disease statistical tests: key significant comparisons MWU: Mann-Whitney U test; KS: Kolmogorov-Smirnov

Gene set	Comparison	n paired	Mean diff	Cohen's d paired	Cohen's d unpaired	Significant test/interpretation
REACTOME_COMPLEMENT_ALL	oex_uex vs oud_oex	26	-1.149	-0.44	-0.78	MWU p=0.01036; Welch p=0.004365; Brunner-Munzel p=0.005634; paired t p=0.03453; paired Wilcoxon p=0.03955; sign-flip permutation p=0.0371
REACTOME_ENERGY_DEPENDENT_REGULATION_OF_MTOR_BY_LKB1_AMPK_ALL	oex_uex vs oud_oex	17	-1.707	-0.62	-0.73	MWU p=0.02012; Welch p=0.02971; Brunner-Munzel p=0.01287; paired t p=0.02186; paired Wilcoxon p=0.01743; sign-flip permutation p=0.0331
REACTOME_SIRT1_NEGATIVELY_REGULATES_RRNA_EXPRESSION_ALL	oex_uex vs oud_uex	6	1.218	1.46	0.49	Paired t p=0.01578; paired Wilcoxon p=0.03125; sign-flip permutation p=0.0302
ADULT_MICRO_ALL	oex_uex vs oud_uex	14	-1.064	-0.50	-0.89	MWU p=0.03035; Welch p=0.02139; Brunner-Munzel p=0.0199
ADULT_MICRO_ALL	oud_oex vs oud_uex	12	-0.663	-0.60	-0.39	Paired Wilcoxon p=0.02441; sign-flip permutation p=0.0449
ADULT_ASTRO_ALL	oud_oex vs oud_uex	19	0.772	0.52	0.46	Paired t p=0.0351; paired Wilcoxon p=0.04456; sign-flip permutation p=0.0371
KEGG_APOPTOSIS_ALL	oex_uex vs oud_oex	47	-0.389	-0.13	-0.26	MWU p=0.03836; KS p=0.04962; Brunner-Munzel p=0.03948
REACTOME_ENERGY_DEPENDENT_REGULATION_OF_MTOR_BY_LKB1_AMPK_ALL	oex_uex vs oud_uex	16	-1.247	-0.56	-0.52	Paired t p=0.04152; sign-flip permutation p=0.0496
ADULT_ASTRO_ALL	oex_uex vs oud_oex	19	-1.086	-0.32	-0.60	Welch p=0.04505; Brunner-Munzel p=0.04586
WP_NAD_METABOLISM_SIRTUINS_AND_AGING_ALL	oex_uex vs oud_uex	10	1.299	0.64	0.73	Effect-size significant
REACTOME_SIRT1_NEGATIVELY_REGULATES_RRNA_EXPRESSION_ALL	oex_uex vs oud_oex	6	1.096	0.60	0.69	Effect-size significant
GOBP_SYNAPSE_PRUNING_ALL	oex_uex vs oud_oex	11	-0.767	-0.33	-0.75	Effect-size significant

**Table 12:** LOO influence: sign-flip gene entries only LOO table restricted to sign-flip entries and high-impact sign-flip examples visible in the provided summaries. Duplicate rows in the follow-up summary were collapsed. LOO: leave-one-out

Gene set	Contrast	Gene	TWAS Z	ΔStouffer	Influence %	Flag
GOBP_CAMKK_AMPK_SIGNALING_CASCADE_ALL	oex_uex	MYH7B	3.29	1.341	14914.8	SIGN-FLIP
GOMF_NADPLUS_BINDING_ALL	oud_oex	SIRT6	3.92	1.085	6185.2	SIGN-FLIP
GOBP_CAMKK_AMPK_SIGNALING_CASCADE_ALL	oex_uex	CAMKK2	1.13	0.462	5137.6	SIGN-FLIP
GOMF_NADPLUS_BINDING_ALL	oud_oex	SIRT5	3.23	0.896	5108.3	SIGN-FLIP
REACTOME_NEUROTRANSMITTER_RELEASE_CYCLE	oud_oex	STX1A	-2.67	-0.843	3410.3	SIGN-FLIP
GOBP_CAMKK_AMPK_SIGNALING_CASCADE_ALL	oex_uex	HTT	0.38	0.155	1730.0	SIGN-FLIP
GOMF_NADPLUS_BINDING_ALL	oud_oex	CRYL1	1.08	0.300	1709.4	SIGN-FLIP
REACTOME_NEUROTRANSMITTER_RELEASE_CYCLE	oud_oex	VAMP2	-1.17	-0.369	1493.9	SIGN-FLIP
GOBP_SYNAPSE_PRUNING_ALL	oud_oex	C1QB	-1.28	-0.40	783.2	SIGN-FLIP
GOBP_SYNAPSE_PRUNING_ALL	oud_oex	CX3CR1	-1.13	-0.36	692.1	SIGN-FLIP
GOMF_NADPLUS_BINDING_ALL	oud_uex	SIRT5	3.59	0.95	566.2	SIGN-FLIP
GOMF_NADPLUS_BINDING_ALL	oud_uex	SIRT6	2.18	0.58	343.0	SIGN-FLIP
HALLMARK_PI3K_AKT_MTOR_SIGNALING_ALL	oud_oex	PLCG1	-4.21	-0.52	795.9	SIGN-FLIP
HALLMARK_PI3K_AKT_MTOR_SIGNALING_ALL	oud_oex	MAP2K3	-4.20	-0.52	794.8	SIGN-FLIP
HALLMARK_PI3K_AKT_MTOR_SIGNALING_ALL	oud_oex	SLA	-3.77	-0.46	713.0	SIGN-FLIP
KEGG_NICOTINATE_AND_NICOTINAMIDE_METABOLISM_ALL	oud_oex	NAMPT	-2.85	-0.66	130.9	SIGN-FLIP
GOBP_CELLULAR_SENESCENCE_ALL	oud_oex	TERF2	2.93	0.40	382.1	SIGN-FLIP
REACTOME_INTRINSIC_PATHWAY_FOR_APOPTOSIS_ALL	oud_oex	CASP7	-5.81	-1.01	249.2	SIGN-FLIP
REACTOME_REGULATION_OF_INSULIN_SECRETION_ALL	oex_uex	ADCY6	5.33	0.77	227.9	SIGN-FLIP
REACTOME_GLUCAGON_LIKE_PEPTIDE_1_GLP1_REGULATES_INSULIN_SECRETION_ALL	oud_oex	GNG10	2.30	0.49	401.4	SIGN-FLIP
REACTOME_GLUCAGON_LIKE_PEPTIDE_1_GLP1_REGULATES_INSULIN_SECRETION_ALL	oud_oex	PRKAR2B	2.18	0.46	379.5	SIGN-FLIP
REACTOME_TELOMERE_ALL	oud_oex	TERF2	2.93	0.45	30.6	SIGN-FLIP
WP_NAD_METABOLISM_ALL	oud_oex	SIRT6	3.92	1.13	53.4	SIGN-FLIP
WP_NAD_METABOLISM_ALL	oud_oex	NAMPT	-2.85	-1.01	47.4	SIGN-FLIP
REACTOME_SYNTHESIS_SECRETION_AND_INACTIVATION_OF_GLP_1_ALL	oud_oex	CTNNB1	-3.86	-1.33	236.6	SIGN-FLIP

**Table 13:** MHC, complement, and immune-gene flagging Immune/MHC-containing gene sets were flagged for interpretive caution. No immune-exclusion re-run was performed in the provided summary. MHC: major histocompatibility complex

Gene set	Genes flagged or removed	Genes
GOBP_INSULIN_SECRETION_INVOLVED_IN_CELLULAR_RESPONSE_TO_GLUCOSE_STIMULUS_ALL	1	HLA-DRB1
GOBP_REGULATION_OF_CELLULAR_SENESCENCE_ALL	2	B2M; HLA-G
GOBP_CELLULAR_SENESCENCE_ALL	2	B2M; HLA-G
MOOTHA_PGC_ALL	1	HLA-DRA
GOBP_INTRINSIC_APOPTOTIC_SIGNALING_PATHWAY_ALL	1	CLU
HALLMARK_APOPTOSIS_ALL	2	CLU; TAP1
SAUL_SEN_MAYO_ALL	2	C3; CD55
GOBP_SYNAPSE_PRUNING_ALL	5	C1QA; C1QB; C1QC; C1QL1; C3
ADULT_MICRO_ALL	3	C3; C3AR1; HLA-DRA
REACTOME_COMPLEMENT_ALL	110	Complement, immunoglobulin, and MHC-related genes including C1QA, C1QB, C1QC, C3, C4A, C4B, C5, CD46, CD55, CD59, CFB, CFH, CFI, CR1, CLU, immunoglobulin heavy-/light-chain genes, MASP1, MASP2, MBL2, PROS1, SERPING1, and VTN

**Table 14:** Gene-set overlap and unique-gene robustness

Gene-set pair	Jaccard overlap	Shared genes, n	Interpretive note
REACTOME_CELLULAR_SENESCENCE_ALL ∩ REACTOME_SENESCENCE_ALL	1.000	197	Complete redundancy
REACTOME_REGULATION_OF_INSULIN_SECRETION_ALL ∩ REACTOME_GLUCAGON_LIKE_PEPTIDE_1_GLP1_REGULATES_INSULIN_SECRETION_ALL	0.538	42	High overlap
GOBP_REGULATION_OF_CELLULAR_SENESCENCE_ALL ∩ GOBP_CELLULAR_SENESCENCE_ALL	0.505	55	High overlap
HALLMARK_INFLAMMATORY_RESPONSE ∩ HALLMARK_TNFA_SIGNALING_VIA_NFKB	0.424	14	Moderate overlap
WP_NAD_METABOLISM_ALL ∩ WP_NAD_BIOSYNTHETIC_PATHWAYS_ALL	0.407	11	Moderate overlap
REACTOME_SIRT1_NEGATIVELY_REGULATES_RRNA_EXPRESSION_ALL ∩ REACTOME_TELOMERE_ALL	0.346	46	Moderate overlap
KEGG_NICOTINATE_AND_NICOTINAMIDE_METABOLISM_ALL ∩ REACTOME_NICOTINATE_METABOLISM_ALL	0.325	13	Moderate overlap
MOOTHA_MITOCHONDRIA_ALL ∩ HALLMARK_OXIDATIVE_PHOSPHORYLATION_ALL	0.292	147	Below 0.30 but many shared genes
REACTOME_SIRT1_NEGATIVELY_REGULATES_RRNA_EXPRESSION_ALL ∩ REACTOME_SENESCENCE_ALL	0.288	59	Below 0.30
REACTOME_CELLULAR_SENESCENCE_ALL ∩ REACTOME_SIRT1_NEGATIVELY_REGULATES_RRNA_EXPRESSION_ALL	0.288	59	Below 0.30
Gene sets retaining ≥3 unique genes	-	45 sets	Supports partial curation robustness

**Table 15:** Core opposing exposure-versus-dependence profile result CCC: concordance correlation coefficient; FDR: false discovery rate; BH-FDR: Benjamini-Hochberg false discovery rate

Metric	Result	Definition	Interpretation
Opposing profiles	100/102	Negative Pearson r, sign concordance <0.5, or Lin's CCC <0.4	Most evaluable ageing-related gene-set profiles were directionally opposed between exposure and dependence
Most opposing Pearson r	-0.938	GOBP_POSITIVE_REGULATION_OF_MICROGLIAL_ACTIVATION, oex_uex vs oud_oex	Strong inverse profile
Most opposing Lin's CCC	-0.865	REACTOME_SYNTHESIS_SECRETION_AND_INACTIVATION_OF_GLP_1_ALL	Strong discordance
Lowest sign-concordance example	0.000	GOBP_NAD_TRANSPORT_ALL	Very small n; interpreted cautiously
Global tissue heterogeneity	0/149	Kruskal-Wallis p<0.05 across tissue decompositions	No significant tissue heterogeneity detected in the follow-up summary
Total p-values in master inventory	4913	All p-values pooled across output tables	Used for single global BH-FDR inventory
Global BH-FDR survivors	131	Global FDR<0.05	Most conservative summary in follow-up package
Global FDR survivors by source	2/66/63	Enrichment/pairwise_stats/proximity	Most global FDR survivors were profile and pairwise/concordance statistics

**Table 16:** Family-wise FDR: top globally significant findings FDR: false discovery rate

Source	Gene set	Test	Comparison or contrast	p	Global FDR
proximity	GOBP_INTRINSIC_APOPTOTIC_SIGNALING_PATHWAY_ALL	Pearson p	oex_uex vs oud_oex	0	0
proximity	GOBP_INTRINSIC_APOPTOTIC_SIGNALING_PATHWAY_ALL	Pearson p	oud_oex vs oud_uex	0	0
proximity	GOBP_INTRINSIC_APOPTOTIC_SIGNALING_PATHWAY_ALL	Spearman p	oud_oex vs oud_uex	0	0
pairwise_stats	GOBP_INTRINSIC_APOPTOTIC_SIGNALING_PATHWAY_ALL	Kendall p	oud_oex vs oud_uex	0	0
proximity	HALLMARK_APOPTOSIS_ALL	Pearson p	oex_uex vs oud_oex	0	0
proximity	HALLMARK_OXIDATIVE_PHOSPHORYLATION_ALL	Pearson p	oud_oex vs oud_uex	0	0
proximity	HALLMARK_OXIDATIVE_PHOSPHORYLATION_ALL	Spearman p	oud_oex vs oud_uex	0	0
pairwise_stats	HALLMARK_OXIDATIVE_PHOSPHORYLATION_ALL	Kendall p	oud_oex vs oud_uex	0	0
proximity	MOOTHA_MITOCHONDRIA_ALL	Pearson p	oex_uex vs oud_uex	0	0
proximity	MOOTHA_MITOCHONDRIA_ALL	Pearson p	oud_oex vs oud_uex	0	0
proximity	MOOTHA_MITOCHONDRIA_ALL	Spearman p	oud_oex vs oud_uex	0	0
pairwise_stats	MOOTHA_MITOCHONDRIA_ALL	Kendall p	oud_oex vs oud_uex	0	0
proximity	MOOTHA_PGC_ALL	Pearson p	oex_uex vs oud_oex	0	0
proximity	MOOTHA_PGC_ALL	Pearson p	oud_oex vs oud_uex	0	0
pairwise_stats	MOOTHA_PGC_ALL	Sign-concordance p	oud_oex vs oud_uex	0	0
enrichment	REACTOME_COMPLEMENT_ALL	Permutation p	oex_uex	0.0007	0.009273
pairwise_stats	REACTOME_COMPLEMENT_ALL	Welch p	oex_uex vs oud_oex	0.004365	0.04278
pairwise_stats	REACTOME_COMPLEMENT_ALL	Brunner-Munzel p	oex_uex vs oud_oex	0.005634	0.04601
proximity	REACTOME_REGULATION_OF_INSULIN_SECRETION_ALL	Pearson p	oex_uex vs oud_oex	0.000872	0.04273
proximity	HALLMARK_INFLAMMATORY_RESPONSE	Pearson p	oud_oex vs oud_uex	0.001894	0.0464
